# Supplementary material for: Extreme transport of light in spheroids of tumor cells
Source: Nat Commun. 2023 Aug 3;14:4662. doi: 10.1038/s41467-023-40379-7 (PMC10400595; doi:10.1038/s41467-023-40379-7)
Supplement: Supplementary file 1 — Supplementary Information [file 41467_2023_40379_MOESM1_ESM.pdf]

# Supplementary Material for: Extreme transport of light in spheroids of tumor cells

Davide Pierangeli[1,2*], Giordano Perini[3,4], Valentina Palmieri[1,3], Ivana Grecco[2], Ginevra Friggeri[3,4], Marco De Spirito[3,4], Massimiliano Papi[3,4*], Eugenio DelRe[2], Claudio Conti[2]

[1]Institute for Complex Systems, National Research Council, Rome, 00185, Italy.
[2]Physics Department, Sapienza University of Rome, Rome, 00185, Italy.
[3]Neuroscience Department, University Cattolica del Sacro Cuore, Rome, 00168, Italy.
[4]IRCSS, Fondazione Policlinico Universitario Agostino Gemelli, Rome, 00168, Italy.

*Corresponding author(s). E-mail(s): davide.pierangeli@roma1.infn.it; massimiliano.papi@unicatt.it;

# Supplementary Figures

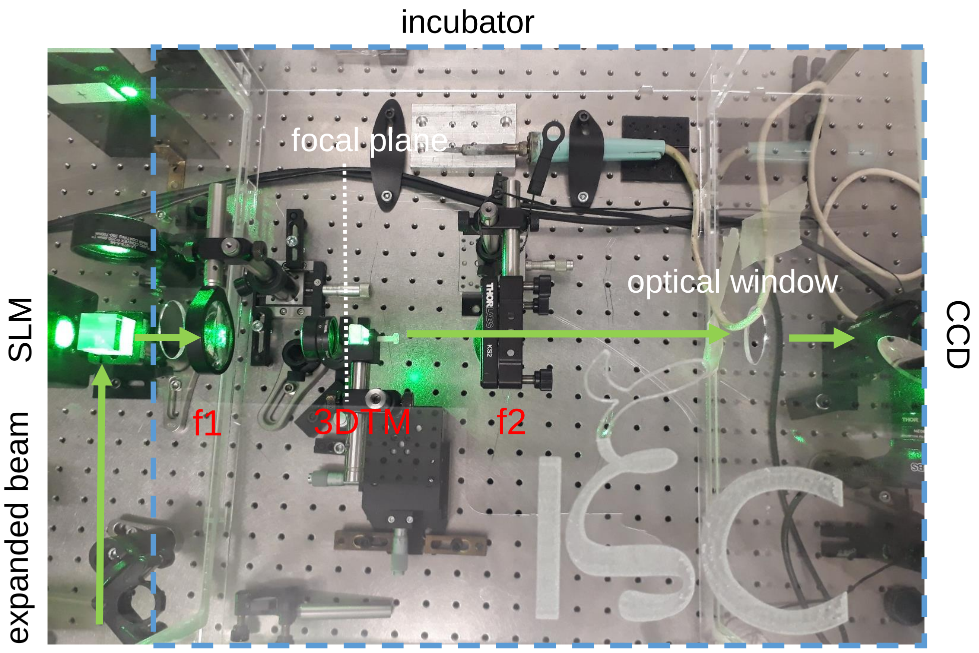


**Supplementary Fig. 1** Photo of the experimental setup (see also Ref. [1]). Shown are: the expanded CW laser beam ($\lambda$ = 532nm), the spatial light modulator (SLM), the focusing and imaging lens (f1=100mm, f2=75mm), the sample holder with the tumor spheroid (3DTM) in the focal plane, and the path toward the camera (CCD). The optical setup is enclosed in a custom-build incubator visible as a glass box with optical windows.

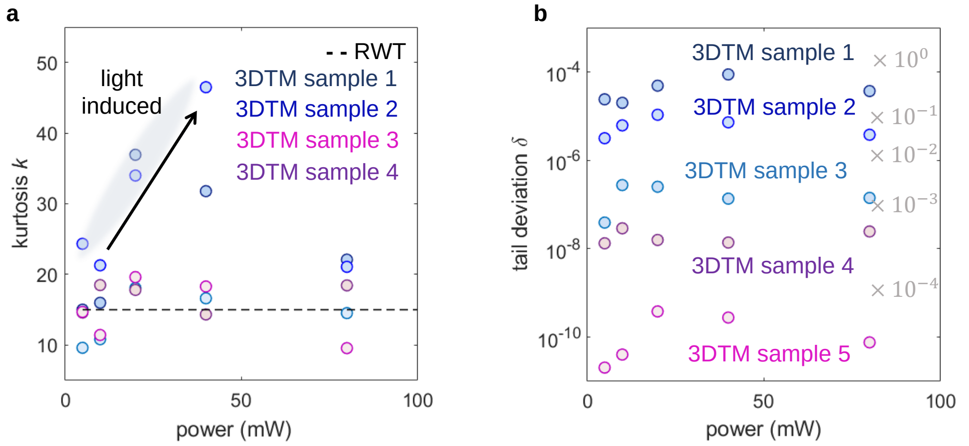


**Supplementary Fig. 2** Statistics of the ransmitted intensity for various 3DTM samples. (a) Measured kurtosis $k$, i.e. the fourth-order moment that quantifies the heaviness of the PDF tail, when varying the input power for several 3DTM samples. The results show moderate sample-to-sample fluctuations of the PDF, i.e., a modest dependence on the local 3DTM structure. Evidenced is the appearance of RWs that is triggered by an increase of the optical power. (b) Power-dependence of the deviation $\delta$ of the statistical tail from the RWT. For clarity, data are vertically shifted by the factors inset. $\delta$ is computed by averaging, within the intensity interval $[I^*, I^*+1]$, with intensity $I^* = 10$, the difference between the observed and RWT density. Errors over three repeated measurements are

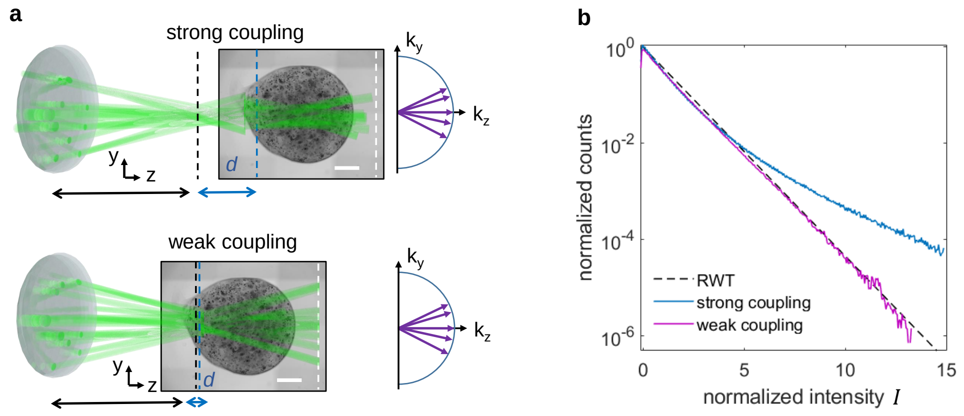


**Supplementary Fig. 3** Control of optical extreme waves appearance via input beam size. (a) Scheme of the optical configuration leading to strong and weak coupling conditions. The insets illustrate that the set of random input wavevectors is the same in both cases. (b) Intensity PDF showing RWs emergence and suppression (normal waves, NW) for strong ($d = 1$ mm) and weak ($d = 0$) coupling, respectively.

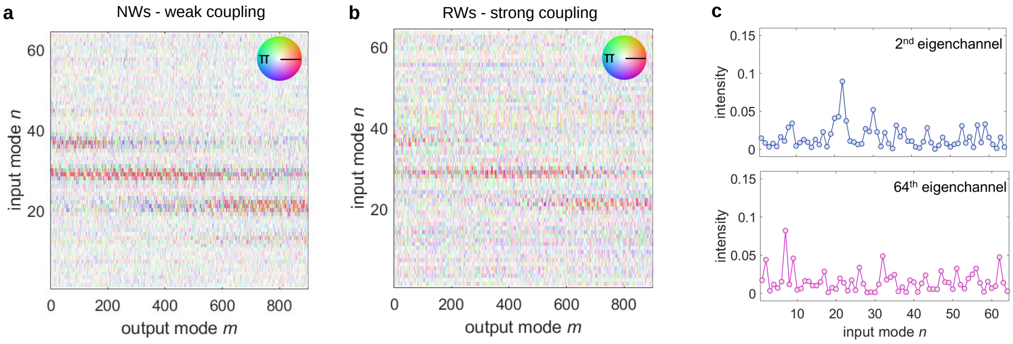


**Supplementary Fig. 4** (a-b) Amplitude and phase plot of the measured transmission matrix (TM) [2] analyzed in Fig. 5 for (a) weak and (b) strong coupling ($N = 64$, $M = 900$, and $P = 60$ mW). (c) Intensity profiles of two exemplary transmission eigenchannels for the RW case in (b).

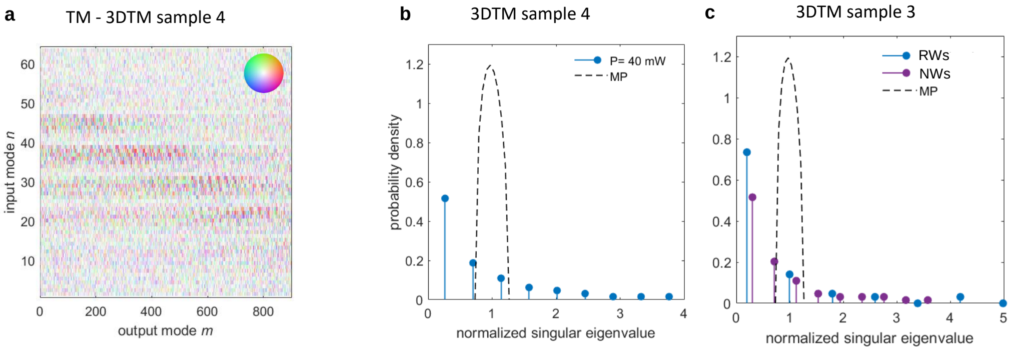


**Supplementary Fig. 5** (a) Complex-valued plot of a 3DTM transmission matrix (TM) ($P = 40$ mW). (b-c) Distribution of the transmission singular eigenvalues $\sqrt{\tau}$ for (b) the TM in (a), and (c) the TM measured on a different 3DTM sample for weak (normal waves, NWs) and strong (rogue waves, RWs) coupling. MP: Marcenko–Pastur law [3].

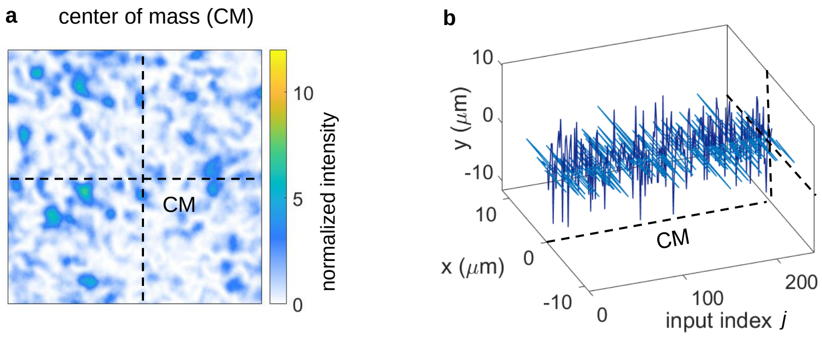


**Supplementary Fig. 6** Analysis of the transmitted speckle intensity. (a) Speckle pattern within a region of interest (ROI) of $200 \times 200 \mu m^2$. Dotted lines show the computed the intensity center of mass (CM). (b) Measured variations of the CM for a set of randomly selected input phase masks.

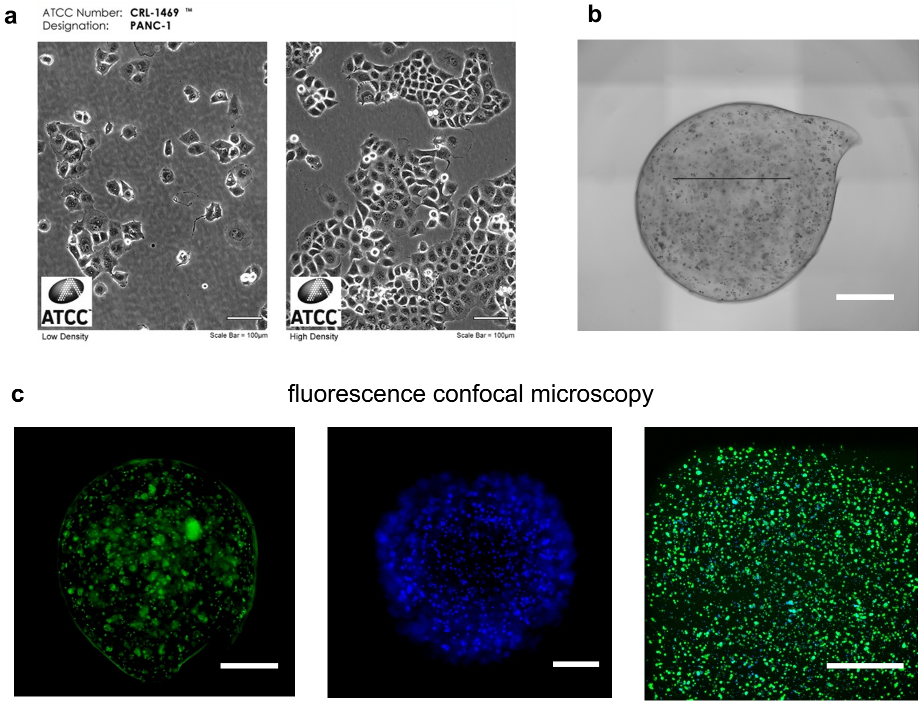


**Supplementary Fig. 7** Microscopy images of 3DTMs. Bright-field microscopy of (a) the cancer cell line PANC-1 employed for the growth of the tumor spheroids and (b) the bio-printed 3DTM. (c) Fluorescence and confocal microscopy of 3DTM stained with Calcein-AM (central image) and 4',6-diamidine-2-fenilindolo (DAPI, right image). Scale bars are 500 $\mu$m.

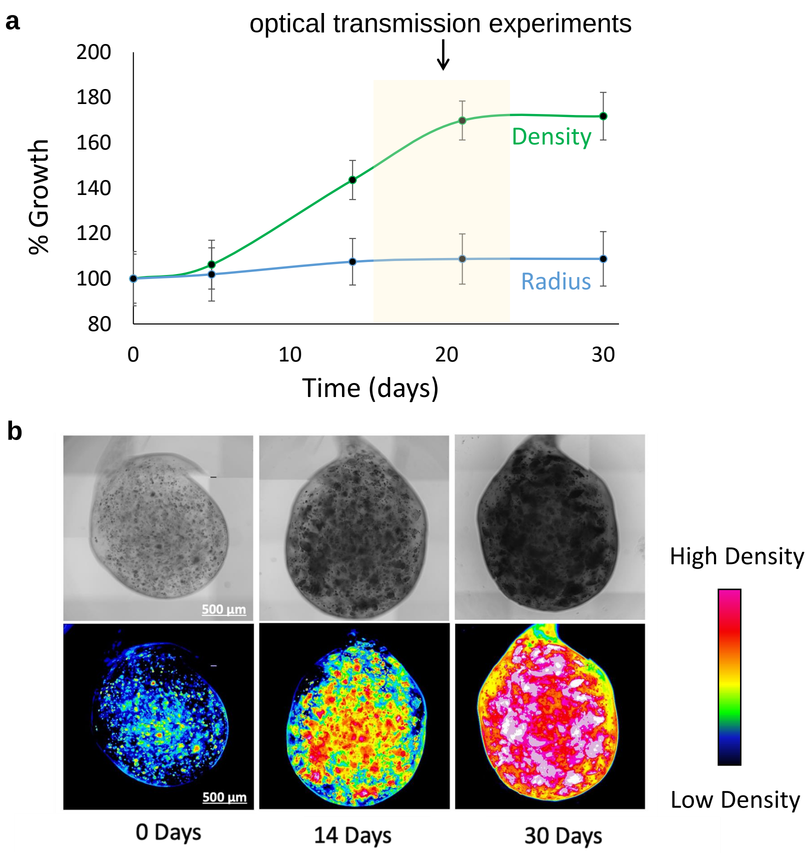


**Supplementary Fig. 8** Evolution of the tumor spheroids. (a) Mean relative variation of the density and radius of the bio-printed 3DTMs as a function of time. Error bar is the standard deviation over 5 biological samples. The shaded area indicates the temporal window in which the optical transmission experiments have been performed. (b) Bright microscopy images of a 3DTM sample as growth and after 14 and 30 days. The image processed via Fiji tool [4] (false-color images) shows an inhomogeneous distribution of the cell clusters, with an increased cell density around the spheroid center due to cell replication.

# Supplementary References

[1] D. Pierangeli, V. Palmieri, G. Marcucci, C. Moriconi, G. Perini, M. De Spirito, M. Papi, and C. Conti, Living optical random neural network with three dimensional tumor spheroids for cancer morphodynamics, *Commun. Phys.* **3**, 160 (2020).

[2] A. Boniface, J. Dong, and S. Gigan, Non-invasive focusing and imaging in scattering media with a fluorescence-based transmission matrix, *Nat. Commun.* **11**, 6154 (2020).

[3] S.M. Popoff, G. Lerosey, M. Fink, A.C. Boccara, and S. Gigan, Controlling light through optical disordered media: transmission matrix approach, *New J. Phys.* **13**, 123021 (2011).

[4] C. Moriconi, V. Palmieri, R. Di Santo, G. Tornillo, M. Papi, G. Pilkington, M. De Spirito, M. Gumbleton, INSIDIA: a FIJI macro delivering high-throughput and high-content spheroid invasion analysis, *Biotechnol. J.* **12**, 1700140 (2017).
